# The functions of securities law: An expert survey of legislative intent in Poland

**DOI:** 10.1371/journal.pone.0246117

**Published:** 2021-01-28

**Authors:** Dawid Van Kędzierski, Paweł Chmielnicki, Michał Stachura, Dobrochna Minich

**Affiliations:** 1 Faculty of Law and Administration, Department of Legal Theory and Material Sources of Law, Lazarski University, Warsaw, Poland; 2 Faculty of Law and Social Sciences, Department of Economics and Finance, Jan Kochanowski University in Kielce, Kielce, Poland; National Institute of Public Finance and Policy, INDIA

## Abstract

Under the conventional view, securities law is intended to protect ordinary (retail) investors. However, some scholars from the school of Law & Economics (L&E), guided by considerations of economic efficiency, claim instead that the principal function of securities law is to reduce transaction costs and risk to professionals. This paper examines those claims empirically. Our research design blends content analysis methods with expert survey techniques to arrive at numerical assessments of determinants of Polish securities law. We find that the L&E view is supported by our data very weakly, only insofar as consumer protection is not the main driver of securities law. The veracity of this claim appears to be time-dependent. We do not find sufficient support for the main components of the L&E view, i.e., that securities law is meant to reduce transaction costs and risk to professionals. However, our data does not refute the L&E hypotheses, and, therefore, we consider them to be an open question. We phrase our conclusions cautiously because of the relatively small number of experts surveyed and of statutory sources of securities law in Poland.

## Introduction

There is a widespread belief that securities law is meant to protect ordinary (retail) investors. That view of securities law was articulated by U.S. lawmakers in the 1930s [1] and upheld by courts in subsequent decades [2,3]. Following the 2008 global financial crisis, considerations of consumer protection were among those that led the U.S. Congress to pass the Dodd–Frank Wall Street Reform and Consumer Protection Act (2010) [4]; a sweeping overhaul of financial regulation extending well beyond the area of securities law. Similar concerns underlay the European Union (EU) Capital Markets Union project. The European Commission stated in no uncertain terms that the financial crisis “shattered” retail investor trust in capital markets, and consumer protection rules are seen to be “instrumental” in restoring that trust within the EU [5] (p. 62). Considerations of protecting retail investors permeate much of EU securities law [6,7]. The rationale for that is the assumption that consumers (retail investors) require additional protection because of having a perceived weakness in relation to professional participants in securities markets. Securities markets are complex, and retail investor experience and knowledge of securities products does not usually match that of professionals’.

A similar perspective underlies the trend of environmental, social, and governance (ESG) investing [8] that has been emerging in recent years on the back of rising concerns over social inequality and climate change. ESG investing is facilitated by the practice of non-financial ESG reporting that is becoming more and more widespread in corporate securities markets [9,10]. Even public sector issuers, such as cities and local governments, are facing calls for greater disclosure of ESG issues [10].

A different perception of the functioning of securities law has developed within the mainstream of the Law & Economics paradigm [11]. Scholars working under this paradigm analyze the law through the lens of economic efficiency in search of legal mechanisms that facilitate the attainment of optimal outcomes of economic activity. In the area of corporate law, this manifests itself in the well-known doctrine of shareholder value maximization [12,13]. In the area of securities law, a widely cited view posits that the main functions of securities law are reducing transaction costs and risk to professional investors rather than protecting consumers [14]. Under that view, securities law should promote efficient markets where prices of securities are accurate and liquidity is high to improve allocation of resources in the economy. We call this the cost-and-risk reduction (CRR) hypothesis, and we use the generic term “functional hypothesis” as a name for any hypothesis concerning the aims, functions or goals of law, or certain parts of law.

There are other functional hypotheses concerning securities law [15,16]. One of them has been refuted by showing that its implications do not hold up empirically [17]. Although we understand the virtue of that approach, we do not find it fully convincing. Functional hypotheses can be understood to be descriptions of the legislative intent (*ratio legis*) behind a law, i.e., the goals that the law was meant to achieve. That interpretation aligns closely with traditional legal scholarship by which *ratio legis* (or teleological) inferences abound [18]. It also carries an important corollary; namely, the falsification of empirical implications of a functional hypothesis proves only that the aim of a law has not been met in reality. It may still be that the hypothesized goals were indeed the reason for enacting the law, but the lawmakers were incompetent in drafting the rules. We based our study on that interpretation of functional hypotheses.

Testing functional hypotheses under that interpretation requires a different research design; one that is intended to expose legislative intent. Because that has not been done previously, this study has a significant exploratory component. Our approach to testing the CRR hypothesis was to operationalize legislative intent by assuming that the “aim of the law” (or its goals) is that that can be attributed to lawmakers from perusal of available parliamentary documents. To further refine our research problem, we divided the CRR hypothesis into three distinct sub-hypotheses:

H1. Securities law does not aim to protect consumers.
H2. Securities law aims to reduce transaction costs for professionals.
H3. Securities law aims to reduce risk to professionals.

For statistical testing of the hypotheses, we have utilized a database created in a two-stage process. The first stage involved processing massive amounts of unstructured text produced from legislative procedures in the Polish parliament to extract relevant data. The second stage was an expert survey of the extracted data to assign numerical scores to permit us to measure the intensity with which certain goals, corresponding to the research questions that guided data-extraction, are pursued by the laws enacted by parliament. The goal of our testing was to compare the sample of individual securities laws to the whole body of statutory law to detect any statistically significant differences.

We must emphasize at the outset that this is an exploratory pilot study due to two factors. First, despite the relatively large number (1079) of all statutes under study, only twelve concerned securities law directly. This small sample size is mainly due to a high concentration of Polish securities law in a relatively small number of statutes, and partial harmonization of securities law at the EU level. Securities law policy, however, remains mainly driven at the national level: most pieces of EU harmonization have the form of directives that need to be transposed into national law (they are not directly applicable), and securities laws, both EU and national, are enforced by national authorities. Second, we have surveyed a somewhat low number (8) of experts. With respect to the expert survey we are following La Porta, Lopez-de-Silanes, and Shleifer [19], the authors of the influential legal origins theory, who developed their theory in part based on a survey of a single expert in every country under study. One of the main reasons for the relatively low number of respondents is that the survey required each expert to expend significant time and cognitive attention. Many potential experts were hesitant to make such commitments.

Before proceeding further, we need to mention that the body of law that is the subject of this study is variously referred to as “securities law” (more often in the U.S.) or “capital markets law” (more often in Europe). The difference is merely terminological, and not substantive.

## Materials and methods

We emphasize that the exercise to collect data was part of a wider program of researching legislative intent. In this paper we only report on three aspects.

Our research protocol divided the study into two stages. The first was reviewing the documents relating to each statute using the methods of content analysis [20]. That enabled us to extract relevant data that were summarized in abstracts. The second stage was an expert survey–a developed method [21] that also finds use in empirical legal research [19]. We note that, in Poland, only medical experiments and clinical trials are subject to approval by ethics committees [22]; since our study is neither, it was not (and could not have been) reviewed by an ethics committee. The data are reported and analyzed such that experts are anonymous, i.e., not identifiable from the results. The nature of the data reported is not of a personal or otherwise sensitive nature.

For the first stage, we analyzed the legislative procedures associated with statutes (*ustawa*) passed by both chambers of the Polish parliament (the *Sejm* and the *Senat*) in 2014–2019. Statutes are a primary source of law in Poland ranking below the constitution in terms of binding power. Statutes were the *target-units*; i.e., the units under analysis [21]. Strictly speaking, we did not sample the statutes and chose instead to analyze the entire corpus of Polish statutory law for the period under investigation. However, for intuition, we will sometimes refer to the set of all statutes, or subsets of it, as “samples” (dropping the quotation marks). The grand total of all analyzed statutes was 1079.

We reviewed all documents produced during the legislative process; most importantly, official substantiations of the bills (*uzasadnienie*), regulatory impact assessments, stenographic records of plenary parliamentary sessions, transcripts of parliamentary committee hearings, and opinions of parliamentary counsel and external experts or organizations, that were invited to opine. Data extracted from all those documents were compiled into separate abstracts (one abstract for each statute). We did not analyze the bills, or enacted statutes. The rationale for that is that statutes consist exclusively of operative clauses that are direct sources of law. The background reasons for adopting those clauses are given in the official substantiation (*uzasadnienie*) accompanying each bill. The substantiation is produced by the government, members of parliament, or the president, depending on who submitted the bill. We were interested in the reasons that were given for proposing and passing securities laws not in the content of those laws. We grouped the reasons into ten categories, given in Table 1. We repeat them in the form that was provided to the experts.

**Table 1:** Research questions for stage one.

Research question	Description
Q1. Protection of the “meek”	Does the regulation strengthen the protection of democratically-respected rights of individuals? For example: • Does it facilitate the exercise of those rights against persons having a political or an economic advantage over the individual? • Does it enhance the instruments used to control those wielding political power? • Does it permit individuals to access information concerning the exercise of state power or to directly make political decisions? • Does it protect employees against employers? • Does it protect consumers against monopolists or oligopolists?
Q2. Transaction costs	Does the regulation increase burdens or difficulties (transaction costs, barriers to entry) for businesses? For example: • Does it necessitate a greater allocation of resources to public liabilities, such as taxes or social security? • Does it require employment of more personnel to fulfill any criteria defined by public authorities? • Does it require additional time to get things done?
Q3. Risk reduction	Does the regulation facilitate the assessment of chances to achieve economic goals? Does an enacted regulation permit a better calculation of opportunities and risks? For example: • Does it improve certainty of commercial transactions? • Does it decrease the risk of business failure because of random chances or competitive pressure? • Does it provide access to additional sources of information? • Does it create a system of state-funded financial guarantees to any sectors of the economy?
Q4. Status benefits	Does the regulation aim to redistribute goods (tangible or non-tangible) based exclusively on membership in a group (national, ethnic, professional, sex, age, etc.) instead of granting it in consideration for work or other effort? This concerns the redistributive role of the state, i.e. distribution of goods created in the state by granting financial resources or services to those who have not made a direct contribution.
Q5. Specialization	Does the regulation narrow down the group of entities who can benefit from certain social or economic institutions? For example: • Does it carve out a new subset of people who get access to new, dedicated solutions? • Does it limit the right to make use of certain institutions to a smaller group of people?
Q6. Strengthening of public authority	Does the regulation serve the interests of public authorities? For example: • Was it created mainly in order to enhance the potential of public authorities? • Are public authorities granted with more power?
Q7. Public expenses	Does the regulation increase the costs of functioning of the state or local governments, including the costs of public services? For example: • Does it require more spending on defence or police forces? • Does it introduce new social benefits? • Does it raise salaries of public sector employees? • Does it require more manpower or time to perform public duties?
Q8. Consolidation of economic dominance	Does the regulation benefit entities that have a large economic potential? For example: • Does it benefit big businesses? • Does it benefit those who have a large market share?
Q9. Free-rider problem	Does the regulation decrease the free-rider problem as far as it is concerned with redistribution of goods? For example: • Does it make it harder to obtain goods to people who would like to do so at another person’s expense? • Does it minimize the Ringelmann effect?
Q10. System complication	Does the regulation introduce more complex (or more complicated) schemas for attaining goals? For example: • Does it require additional procedures? • Does it introduce new formalities?

Only the first three research questions are relevant for this paper. While they do not perfectly match our hypotheses, we consider the Q1-H1, Q2-H2, Q3-H3 match to be sufficient for the goals of the study. Out of all the other questions, Q10. System complication is perhaps most closely related to our sub-hypotheses. For example, imposing complex compliance obligations on professional investors would likely fall under Q10. This, however, would not be an issue, since the experts have been instructed that the questions are not mutually exclusive. New compliance obligations would also fall under Q2. Transaction costs, and hence would be scored in both overlapping categories. In the other direction, there are no relevant issues in questions 4–10 that would fall outside the scope of questions 1–3.

For the second stage we conducted an expert survey. This method permitted us to explore topics that might otherwise be impossible to study in a systematic fashion, test hypotheses that had previously been untestable, and collect data for difficult-to-measure phenomena [21]. The experts were provided the abstracts that were produced during stage one and requested to score each statute on seven-point Likert-type scales. The Likert-type scales were used to score each statute in terms of each of the ten questions used during stage one (described in Table 1).

All of the experts were academic legal scholars affiliated with three universities (a professor, three post-docs, and four post-graduate research assistants) who have degrees in law or dual degrees in law and economics or law and political science. Four experts (two post-docs and two post-graduate research assistants) are also qualified attorneys with varying extent of professional experience in legal practice. We found that this make-up of the expert team is advantageous due to a number of reasons. First, legal scholars have experience with understanding and reading legal texts and tend to be more objective in their assessments of securities laws than, e.g., lawmakers or securities markets participants. Second, having experts of different seniority levels improves the representativity of scores. Third, attorneys bring a valuable practical view on legal issues. And finally, holders of non-legal degrees are able to judge the law from different perspective. We believe that this has allowed us to gather scores that are accurate and replicable.

The Likert-type scales were not of the typical “agree/disagree” type. Instead we were interested in measuring the scope and reach of the statutory rules. The scores were labeled by having that consideration in mind: absolute values of scores measure the intensity with which the lawmakers tried to affect each of the social and economic phenomena in which we were interested. The choice of positive/negative for scores was determined by the degree of effect of the law (e.g., a positive score for Q1. Protection of the “meek” indicated that the law was meant to protect individuals, while a negative score indicated that the law was meant to lower that protection). The scales are symmetric, but not “balanced” in the sense of having equal intervals at each score increment. The scales are described in Table 2.

**Table 2:** Likert-type scales for stage two.

Score	Description
-3	There is no doubt as to the negative relation between the statute and research question; the statute has significant effect on a wide range of subjects.
-2	There is no doubt as to the negative relation between the statute and research question; the statute has either significant effect on a narrow range of subjects, or limited effect on a wide range of subjects.
-1	There is no doubt as to the negative relation between the statute and the research question; the statute has limited effect on a narrow range of subjects.
0	Either the statute is not connected to the research question, or the connection is indirect or ambiguous, or both.
1	There is no doubt as to the positive relation between the statute and the research question; the statute has limited effect on a narrow range of subjects.
2	There is no doubt as to the positive relation between the statute and the research question; the statute has either significant effect on a narrow range of subjects, or limited effect on a wide range of subjects.
3	There is no doubt as to the positive relation between the statute and the research question; the statute has significant effect on a wide range of subjects.

We chose to implement a mixed multiple-expert/multiple-target design for which every target-unit (statute) was scored by multiple experts. The advantages of the design are well-known, and help to reduce the effect of individual-level biases and increase the reliability of the overall scores on the Likert-type scale (the target-unit measure of the study) [21]. The mapping of experts to statutes (grouped into sets indexed by year in which the statutes were enacted) is shown in Table 3. The year 2016 was not included because securities laws were not enacted.

**Table 3:** Mapping of experts to statutes.

Year	Expert 1	Expert 2	Expert 3	Expert 4	Expert 5	Expert 6	Expert 7	Expert 8
**2014**	X		X		X	X		X
**2015**	X		X		X	X		X
**2017**	X	X		X			X	
**2018**	X			X		X	X	X
**2019**	X			X		X	X	X

One particular challenge we had to face was the impact of random errors, at the level of individual experts, on Likert scores. This challenge can manifest itself in at least two ways. First, “asking experts to rate many different target-units could tax the limits of their expertise and lead to greater random error in individual scores of target-units.” [21] This can lead to heterogeneity in the reliability of data for some statutes. To limit the issue, we have labelled the neutral score (0) in such a way as to encourage experts to focus opining on statutes with which they are most familiar (see Table 2). Ambiguity as to the aims of a statute was generally resolved in favor of the neutral score to permit experts to conserve cognitive resources to accurately score outliers.

Second, it is known that in small pools of experts individual errors can produce an invalid ordering of data [21]. For our study, the gravest concern was the possibility of obtaining inconsistent scores, i.e., negative scores from some experts, and positive scores from others, in relation to the same question/statute combination. We sought to alleviate this problem by drawing from the method of focus groups. After a preliminary scoring exercise, the experts assigned to score the same statutes were invited to a meeting where they were encouraged to discuss any “hard” or “unusual” cases. It happened that inconsistencies in scores were few and mostly resulted either from differences in interpretation that could be overcome, or from additional background knowledge of some experts. In this sense, these focus groups functioned as a quality control measure for maintaining data integrity. After the meeting, the experts were asked to submit final scores.

We have not managed to ensure an identical number of expert scores in relation to each statute. Statutes enacted in 2017 were scored by 4 experts, while statutes enacted in other years were scored by 5 experts (see Table 3). To avoid incomparability because of that factor we had to assign one average score to each statute. The scores were given on Likert-type scales and a natural choice for such an average is the median because of the ordinal level of measurement. However, we were interested in measuring the intensity of the effect of the law and a median gives less weight to outliers. We were, therefore, lead to computing the arithmetic mean. That choice was additionally supported by our research design which encouraged neutral scores; using a measure that is more sensitive to outliers means that whenever an expert held a strong opinion on a matter, the opinion of the expert was given more weight than the opinion of an expert who rated that matter as being ambiguous (possibly because of insufficient expertise in the subject matter of the statute). To arrive back at our Likert-type scales, arithmetic means were rounded. At the end, the reported individual scores can be intuitively interpreted as being given by a “representative” expert.

We are well aware of the controversies surrounding different levels of measurement which includes some authors’ reluctance to even compute expected values and standard deviations for ordinal data [23] (p. 242–246). To be conservative, for primary analyses, we have limited ourselves to computing the arithmetic means of scores for each year and for each of the securities statutes (in addition to the arithmetic means described in the preceding paragraph). As recommended in the literature [24], we have not used those means for further calculations; instead, in our primary tests we have replaced arithmetic means with ranks. Only after those primary tests had been completed did we triangulate by performing tests that do not fully respect the ordinal level of measurement in the data.

In order to perform statistical tests, we had to identify statutes that relate specifically to securities law, out of the grand total of 1079 statutes. The procedure was straightforward because of the high concentration of Polish securities law in a relatively small number of statutes. The statutes branched off into separate amending acts that were easy to trace. We started by identifying all statutes that govern securities law in Poland. These are:

1. *Ustawa z dnia 29 lipca 2005 r*. *o ofercie publicznej i warunkach wprowadzania instrumentów finansowych do zorganizowanego systemu obrotu oraz o spółkach publicznych (t*.*j*. *Dz*. *U*. *z 2019 r*. *poz*. *623*, *ze zm*.*)* [Act on Public Offering and Publicly Listed Companies]
2. *Ustawa z dnia 29 lipca 2005r*.*o obrocie instrumentami finansowymi (t*.*j*. *Dz*. *U*. *z 2020 r*. *poz*. *89)* [Act on Trading in Financial Instruments]
3. *Ustawa z dnia 29 lipca 2005 r*. *o nadzorze nad rynkiem kapitałowym (t*.*j*. *Dz*. *U*. *z 2019 r*. *poz*. *1871*, *ze zm*.*)* [Act on Capital Markets Supervision]
4. *Ustawa z dnia 27 maja 2004 r*. *o funduszach inwestycyjnych i zarządzaniu alternatywnymi funduszami inwestycyjnymi* (t.j. Dz. U. z 2020 r. poz. 95) [Act on Investment Funds and Alternative Investment Fund Management]
5. *Ustawa z dnia 15 stycznia 2015 r*. *o obligacjach (t*.*j*. *Dz*. *U*. *z 2018 r*. *poz*. *483*, *ze zm*.*)* [Act on Bonds]
6. *Ustawa z dnia 29 sierpnia 1997 r*. *o listach zastawnych i bankach hipotecznych (t*.*j*. *Dz*. *U*. *z 2016 r*. *poz*. *1771*, *ze zm*.*)* [And on Covered Bonds and Mortgage Banks]

Of course, some parts of securities law are incidentally governed by other statutes; however, their scope is limited and omitting them did not affect the comprehensiveness of our analysis, as discussed below.

Five of the six statutes indicated above have been originally enacted before 2014 (the starting date of our study). Most of the statutes that we have analysed were, in fact, amendments to that legislation. These amendments usually introduced comprehensive changes to all statutes governing relevant parts of securities law. This means that even statutes omitted from the list above were still captured in our analysis, as long as they were amended together with any one of the six listed acts.

Our procedure involved tracing all statutes that amended the six statutes listed above. This scope of enquiry proved to be too wide: we have recovered some statutes that changed other parts of law, and only marginally affected securities laws. Therefore, we have omitted:

- statutes in which changes to securities law were incidental (for example, there was a change in definitions in other acts and the definitions were used in securities laws);
- statutes whose overriding goal was to change other areas of law (for example, new GDPR-compliant personal data protection laws); and
- statutes that consolidated legislation without changing the substance of the law.

After removing these statutes from the pool, we were left with the following twelve statutes (numbers are codes used in S1 Data):

139. *Ustawa z dnia 5 grudnia 2014 r*. *o zmianie ustawy o obrocie instrumentami finansowymi oraz niektórych innych ustaw (Dz*. *U*. *z 2015 r*. *poz*. *73)*. This amendment concerned mainly short-selling of securities.
157. *Ustawa z dnia 15 stycznia 2015 r*. *o obligacjach (Dz*. *U*. *z 2015 r*. *poz*. *238)*. This act governs the issuance of bonds under Polish law.
214. *Ustawa z dnia 12 czerwca 2015 r*. *o zmianie ustawy o nadzorze nad rynkiem kapitałowym oraz niektórych innych ustaw (Dz*. *U*. *z 2015 r*. *poz*. *1260)*. This act concerned mainly the maintenance costs of capital markets architecture (stock exchange and securities depository).
274. *Ustawa z dnia 24 lipca 2015 r*. *o zmianie ustawy o listach zastawnych i bankach hipotecznych oraz niektórych innych ustaw (Dz*. *U*. *z 2015 r*. *poz*. *1259)*. This act altered the regulation of covered bonds in order to make them a more attractive asset class.
546. *Ustawa z dnia 26 stycznia 2017 r*. *o zmianie ustawy o ofercie publicznej i warunkach wprowadzania instrumentów finansowych do zorganizowanego systemu obrotu oraz o spółkach publicznych (Dz*. *U*. *z 2017 r*. *poz*. *452)*. This act removed mandatory tender offers for buying 5%/10% of all outstanding shares in a publicly listed company.
555. *Ustawa z dnia 10 lutego 2017 r*. *o zmianie ustawy o obrocie instrumentami finansowymi oraz niektórych innych ustaw (Dz*. *U*. *z 2017 r*. *poz*. *724)*. This act was made to ensure compliance with the EU Market Abuse Regulation.
567. *Ustawa z dnia 9 marca 2017 r*. *o zmianie ustawy o obrocie instrumentami finansowymi oraz niektórych innych ustaw (Dz*. *U*. *z 2017 r*. *poz*. *791)*. This act simplified the structure of regulated securities markets and introduced derivatives accounts.
614. *Ustawa z dnia 8 czerwca 2017 r*. *o zmianie ustawy o obligacjach (Dz*. *U*. *z 2017 r*. *poz*. *1199)*. This act introduced additional types of bonds that can be issued by financial institutions in order to fulfil capital requirements.
733. *Ustawa z dnia 1 marca 2018 r*. *o zmianie ustawy o obrocie instrumentami finansowymi oraz niektórych innych ustaw (Dz*. *U*. *z 2018 r*. *poz*. *685)*. This act implemented the EU Markets in Financial Instruments Directive II into Polish law.
860. *Ustawa z dnia 9 listopada 2018 r*. *o zmianie niektórych ustaw w związku ze wzmocnieniem nadzoru nad rynkiem finansowym oraz ochrony inwestorów na tym rynku (Dz*. *U*. *z 2018 r*. *poz*. *2243)*. This act legally restructured the financial supervision authority.
944. *Ustawa z dnia 15 marca 2019 r*. *o zmianie ustawy o nadzorze nad rynkiem finansowym oraz niektórych innych ustaw (Dz*. *U*. *z 2019 r*. *poz*. *875)*. This act concerned mainly securitization of assets.
1073. *Ustawa z dnia 16 października 2019 r*. *o zmianie ustawy o ofercie publicznej i warunkach wprowadzania instrumentów finansowych do zorganizowanego systemu obrotu oraz o spółkach publicznych oraz niektórych innych ustaw (Dz*. *U*. *2019 r*. *poz*. *2217)*. This act implemented the EU Shareholders Rights Directive II into Polish law.

## Results

Tables 4–6 indicate the frequency distributions and mean expert scores for statutes reviewed for the study. Each Table represents a separate research question (described in Table 1). The total number of scores for each research question in a year is equal to the number of statutes passed by parliament during that year. Full data containing scores at the expert level (before taking the arithmetic mean) is available in supplementary material S1 Data.

**Table 4:** Frequency distribution and means for Q1. Protection of the “meek”.

Year	-3	-2	-1	0	1	2	3	Total number of scores/statutes	Mean score
	Number of individual scores								
2014	1	4	9	70	23	37	2	146	0.5685
2015	0	5	6	137	44	39	3	234	0.4915
2016	1	7	5	93	19	28	11	164	0.5244
2017	1	9	8	76	31	37	6	168	0.5595
2018	0	6	4	110	32	40	5	197	0.5635
2019	0	6	1	97	10	50	6	170	0.6765

**Table 5:** Frequency distribution and means for Q2. Transaction costs.

Year	-3	-2	-1	0	1	2	3	Total number of scores/statutes	Mean score
	Number of individual scores								
2014	0	17	18	74	20	17	0	146	0.0137
2015	0	3	30	160	30	11	0	234	0.0684
2016	1	15	12	96	21	16	3	164	0.1037
2017	1	12	26	80	28	18	3	168	0.1190
2018	1	14	20	130	19	11	2	197	-0.0203
2019	1	9	11	106	24	19	0	170	0.1765

**Table 6:** Frequency distribution and means for Q3. Risk reduction.

Year	-3	-2	-1	0	1	2	3	Total number of scores/statutes	Mean score
	Number of individual scores								
2014	0	3	10	103	20	10	0	146	0.1644
2015	0	3	6	159	51	15	0	234	0.2949
2016	0	5	7	106	30	15	1	164	0.2805
2017	0	8	11	80	46	20	3	168	0.4048
2018	0	2	4	139	24	26	2	197	0.3756
2019	0	1	1	121	25	22	0	170	0.3882

For data concerning individual securities statutes we chose not to round the arithmetic means of individual expert scores because of the small number of statutes. Instead, we used unrounded numbers which are more comparable to the full-year means reported in Tables 4–6. Table 7 indicates the means for securities statutes. The statutes are indicated by code-number (consistent with S1 Data).

**Table 7:** Arithmetic means for securities statutes.

Period	Year	Statute	Q1 score	Q2 score	Q3 score
**A**	2014	139	0	1.2	0.2
	2015	157	0	-0.6	1.2
	2015	214	0	2.4	0.4
	2015	274	0	2.2	2
**B**	2017	546	0	-1.5	-1.5
	2017	555	0.5	0	-0.75
	2017	567	0	-1	0
	2017	614	0	-0.25	1.5
**C**	2018	733	0.8	1.2	1.4
	2018	860	0	0	1.6
	2019	944	1.2	1	1.2
	2019	1073	2	0.8	1.8

It is immediately apparent that we cannot treat the scores for individual securities statutes as a time series. Not only are there various numbers of statutes in different years; 2016 is a gap year when parliament did not enact securities laws. They can, however, be cleanly divided into three distinct periods A, B, and C, while still keeping the ordering implied by year of adoption; in fact, the ordering A<B<C is now strict. We will make use of this happenstance later.

Initially, we wanted to check whether the individual securities statute scores, on the one hand, and the full-year scores, on the other, follow the same probability distribution. To do this, we used the binary Wald-Wolfowitz runs test (reported in Table 8). This non-parametric test requires a two-valued data sequence. In our test, the data sequence for each research question was constructed using the following procedure. First, we took the scores for individual securities statutes (from Table 7) and coupled them with the full-year mean scores (from Tables 4–6) corresponding one-to-one with the year of the individual securities statutes. Whenever there were multiple securities statutes in a given year, the full-year scores were duplicated. We then ordered the scores into a monotonically increasing sample, as shown in the column “Score” of Table 8. Second, we labelled each individual securities statute score as “0” and each full-year score as “1”, producing a string of 0s and 1s, as shown in the column “Runs” of Table 8. There are three such strings, each corresponding to a research question. Each of those strings served as the data sequence for the Wald-Wolfowitz runs test. A *run* of a sequence is a maximal non-empty subsequence consisting of equal elements (for example, in the string “00110” there are three runs, “00”, “11” and “0”). If the number of runs is lower than the critical value, we can conclude that the two tested samples have different probability distributions. This can be intuitively explained as follows: whenever the values from two samples are highly “mixed” in the combined ordered sample, the runs are short, so there must be a high number of runs. This corresponds to the situation where both samples follow the same probability distribution. A low number of runs, on the other hand, corresponds to the situation where both samples do not follow the same probability distribution. Our test was one-sided and the critical values in Table 8 were computed at significance level *α* = 0.05.

**Table 8:** Wald-Wolfowitz runs test (no periodization).

Q1		Q2		Q3	
Score	Runs^a^	Score	Runs^a^	Score	Runs^a^
0	0	-1.5	0	-1.5	0
0	0	-1	0	-0.75	0
0	0	-0.6	0	0	0
0	0	-0.25	0	0.1644	1
0	0	-0.0203	1	0.2	0
0	0	-0.0203	1	0.2949	1
0	0	0	0	0.2949	1
0	0	0	0	0.2949	1
0.4915	1	0.0137	1	0.3756	1
0.4915	1	0.0684	1	0.3756	1
0.4915	1	0.0684	1	0.3882	1
0.5	0	0.0684	1	0.3882	1
0.5595	1	0.1190	1	0.4	0
0.5595	1	0.1190	1	0.4048	1
0.5595	1	0.1190	1	0.4048	1
0.5595	1	0.1190	1	0.4048	1
0.5635	1	0.1765	1	0.4048	1
0.5635	1	0.1765	1	1.2	0
0.5685	1	0.8	0	1.2	0
0.6765	1	1	0	1.4	0
0.6765	1	1.2	0	1.5	0
0.8	0	1.2	0	1.6	0
1.2	0	2.2	0	1.8	0
2	0	2.4	0	2	0
**Number of runs**	5*		7*		5*
**Critical value (5%-quantile)**^**b**^	< 9		< 9		< 9

* Significant at the 5% level.

^a^ 1 = scores for full years; 0 = scores for individual securities statutes.

^b^ Critical value was calculated in R using the “qruns” function in the “randtests” package.

Based on the results reported in Table 8, we infer with high confidence that the sequence is not truly random, and individual securities statute scores (the 0s) follow a different probability distribution than full-year scores (the 1s).

Unfortunately, this test did not provide any guidance concerning the directional difference between the two samples. Taking Q1. Protection of the “meek” as an example, is the difference attributable to the fact that securities laws are more consumer-friendly than the whole body of law, or the opposite? We investigated by comparing the difference between scores. For each question, we again took the scores of individual statutes and the corresponding full-year scores (duplicating where necessary). The values of the scores were then replaced with ranks, with higher ranks representing lower scores. As an example, for Q1, the individual statute No. 1073 has the lowest rank because it had the highest score among all individual statutes and corresponding full-year scores. Whenever there were multiple identical scores, they were assigned the arithmetical average of ranks ranging over all such *ex aequo* scores. We have replaced scores with ranks in order to respect the ordinal level of measurement on our Likert-type scales. We then compared the difference between the average rank of the sample individual statutes and the average rank of full-year scores. A sufficiently small difference in average ranks would indicate that there is no statistically significant difference in probability distributions. The test is summarized in Table 9.

**Table 9:** Average score rank difference test (no periodization).

Year	Statute	Q1 score rank		Q2 score rank		Q3 score rank	
		Full year	Individual statutes	Full year	Individual statutes	Full year	Individual statutes
2014	139	6	20.5	16	3.5	21	20
2015	157	15	20.5	14	22	18	6.5
2015	214	15	20.5	14	1	18	12
2015	274	15	20.5	14	2	18	1
2017	546	10.5	20.5	10.5	24	9.5	24
2017	555	10.5	13	10.5	17.5	9.5	23
2017	567	10.5	20.5	10.5	23	9.5	22
2017	614	10.5	20.5	10.5	21	9.5	4
2018	733	7.5	3	19.5	3.5	15.5	5
2018	860	7.5	20.5	19.5	17.5	15.5	3
2019	944	4.5	2	7.5	5	13.5	6.5
2019	1073	4.5	1	7.5	6	13.5	2
**Average score rank**		9.75	15.25	12.8333	12,1667	14.25	10.75
**Average score rank difference (absolute)**		5.5*		0.6667		3.5	
**Critical value (5%-quantile)**^a^		> 4.8333					

* Significant at the 5% level.

^a^ Critical value was estimated by simulation in R. Successive natural numbers from 1 to 24 were divided randomly into two groups of size 12. Average ranks were calculated for both groups and the lesser number was subtracted from the greater. Number of simulation repetitions (10,000,000) permitted the calculation of a 5%-quantile from a simulated distribution of differences in average ranks.

This time we have only detected statistically significant (one-sided test at significance level *α* = 0.05) results with respect to Q1, where the difference between average score ranks is 5.5, i.e. higher than the critical value of 4.8333. Because higher ranks represent lower scores, we have some evidence to support the belief that the H1 subcomponent of the CRR hypothesis could be true; i.e., that securities law does not aim to protect consumers. On average, securities statutes had lower consumer protection scores, as evidenced by the higher average rank of 15.25, compared to 9.75 for the full-year scores. However, the evidence did not permit rejection of the null hypothesis with respect to subcomponents H2 and H3 of the CRR hypothesis. The differences in average ranks equal to 0.6667 (Q2) and 3.5 (Q3) are not high enough to warrant a statistically significant inference.

The results reported so far do not fully account for the temporal features of the data. The statutes were adopted over a span of 6 years and the results may not hold across the whole timespan. To reintroduce the dimension of time into the results, we made use of the periodization described in Table 7. We simply repeated the two previous tests and comparisons were made within periods A, B, and C. Tables 10 and 11 give the results of those tests. The values in Tables 10 and 11 were indicated by analogy to Tables 8 and 9, respectively.

**Table 10:** Wald-Wolfowitz runs test (periodized).

Period	Q1		Q2		Q3	
	Score	Runs	Score	Runs	Score	Runs
**A**	0	0	-0.6	0	0.1644	1
	0	0	0.0137	1	0.2000	0
	0	0	0.0684	1	0.2949	1
	0	0	0.0684	1	0.2949	1
	0.4915	1	0.0684	1	0.2949	1
	0.4915	1	1.2	0	0.4	0
	0.4915	1	2.2	0	1.2	0
	0.5685	1	2.4	0	2	0
**B**	0	0	-1.5	0	-1.5	0
	0	0	-1	0	-0.75	0
	0	0	-0.25	0	0	0
	0.5	0	0	0	0.4048	1
	0.5595	1	0.1190	1	0.4048	1
	0.5595	1	0.1190	1	0.4048	1
	0.5595	1	0.1190	1	0.4048	1
	0.5595	1	0.1190	1	1.5	0
**C**	0	0	-0.0203	1	0.3756	1
	0.5635	1	-0.0203	1	0.3756	1
	0.5635	1	0	0	0.3882	1
	0.6765	1	0.1765	1	0.3882	1
	0.6765	1	0.1765	1	1.2	0
	0.8	0	0.8	0	1.4	0
	1.2	0	1	0	1.6	0
	2	0	1.2	0	1.8	0
**Period**	**Number of runs**					
**A**	2*		3		4	
**B**	2*		2*		3	
**C**	3		4		2*	
**Critical value (5%-quantile)**^b^	< 3					

* Significant at the 5% level.

^a^ 1 = scores for full years; 0 = scores for individual securities statutes.

^b^ Critical value was calculated in R using the “qruns” function in the “randtests” package.

**Table 11:** Average score rank difference test (periodized).

Period	Year	Statute	Q1 score rank		Q2 score rank		Q3 score rank	
			Full year	Individual statutes	Full year	Individual statutes	Full year	Individual statutes
A	2014	139	1	6.5	7	3	8	7
	2015	157	3	6.5	5	8	5	2
	2015	214	3	6.5	5	1	5	3
	2015	274	3	6.5	5	2	5	1
B	2017	546	2.5	7	2.5	8	3.5	8
	2017	555	2.5	5	2.5	5	3.5	7
	2017	567	2.5	7	2.5	7	3.5	6
	2017	614	2.5	7	2.5	6	3.5	1
C	2018	733	6.5	3	7.5	1	7.5	3
	2018	860	6.5	8	7.5	6	7.5	2
	2019	944	4.5	2	4.5	2	5.5	4
	2019	1073	4.5	1	4.5	3	5.5	1
		**Period**						
**Average score rank**		**A**	2.5	6.5	5.5	3,5	5,75	3,25
		**B**	2.5	6.5	2.5	6,5	3,5	5,5
		**C**	5.5	3.5	6	3	6,5	2,5
**Average score rank difference (absolute)**		**A**	4*		2.5		2	
		**B**	4*		2		4*	
		**C**	2		4*		3	
**Critical value (5%-quantile)**^a^			> 3					

* Significant at the 5% level.

^a^ Critical value was estimated by simulation in R. Successive natural numbers from 1 to 8 were divided randomly into two groups of size 4. Average ranks were calculated for both groups and the lesser number was subtracted from the greater. Number of simulation repetitions (10,000,000) permitted the calculation of a 5%-quantile from a simulated distribution of differences in average ranks.

An interesting observation is that significant (*α* = 0.05) results across both Tables concern the same cells with respect to Q1, i.e. both tests provided significant results for periods A and B. As regards period C, the results are not that clear. The Wald-Wolfowitz runs test provides a borderline result for period C. The result is borderline because intervals produced by quantile cuts are mathematically open at critical values, i.e., they do not include the critical value itself. In this case, the critical value was *cv* = 3, and therefore we are not able to tell with full certainty whether a result of 3 runs, as is the case with Q1 period C, is statistically significant at the *α* = 0.05 level. With a larger sample size, we might have been able to determine with greater confidence whether our findings are robust to periodization. The test reported in Table 11, on the other hand, is unequivocal for period C. The difference of average score ranks equal to 2 indicates that there is no statistically significant difference in period C. The results reported previously in Tables 8 and 9, therefore, failed to fully replicate once periodization had been introduced.

As for Q2 and Q3, the tests yielded significant results only for one period for each of those questions; certainly not enough to reject the null. Moreover, the tests provide divergent results–for transaction costs (Q2), the Wald-Wolfowitz test indicates period B as significantly different, while the rank difference test indicates period C; and for risk reduction (Q3), the Wald-Wolfowitz test indicates period C as significantly different, while the rank difference test indicates period B. We thus have no reason to believe that securities statutes, when compared to all statutes under study, are significantly dissimilar with respect to transaction costs and risk reduction.

To gain additional insight, at this point we relax the restrictions placed by the ordinal level of measurement on the use of arithmetic means. This has allowed us to perform three additional tests that take into account paired observations within the data. Namely, we have performed Fisher’s sign test, Friedman’s test and the Wilcoxon signed-rank test (all in Statistica). Fisher’s sign test and Friedman’s test yielded wholly insignificant results that give us no reason to reject the null hypothesis. Because the tests are standard, we do not report the results, but we do take them into account in the conclusions; we report only the results of the Wilcoxon signed-rank test. It produced a p-value of *p*≈0.068 for certain periods; this fails to meet our significance level of *α* = 0.05. See Table 12 for full results of the test. Unlike the tests reported previously, this test yields somewhat significant (*α* = 0.068) results in relation to two periods for all questions corresponding to the subcomponents of the CRR hypothesis. Interestingly, for the consumer protection scores (Q1), the periods indicated as somewhat significant (A and B) overlap with the periods detected as significant by both of our primary periodized tests (in Tables 10 and 11).

**Table 12:** Wilcoxon signed-rank test (periodized).

Pair of variables^a^	Period	p-value
**Q1 pairs**	A	0,0679
	B	0,0679
	C	0,4652
**Q2 pairs**	A	0,1441
	B	0,0679
	C	0,0679
**Q3 pairs**	A	0,0679
	B	0,2733
	C	0,0679

^a^ Pairs consist of scores for full years and scores for individual statutes.

## Discussion

Our results provide partial support for the CRR hypothesis. We can, based on unperiodized tests, reject the null hypothesis only with respect to the H1 subcomponent. Polish securities law seems to be less consumer-centric than the whole body of statutory law. Perhaps other areas of Polish law are more paternalistic with respect to consumers than is securities law.

However, the results concerning H1 are not robust across different time periods; in period C, there has been an uptick in consumer protection in securities law. We suspect that one of the main reasons behind that is the GetBack corporate scandal that has been ongoing since 2018 [25,26], which is the precise year when the last data period C begins. Over nine thousand investors, mostly individuals, invested around PLN 2.6 billion in GetBack bonds. GetBack defaulted amid allegations of fraud, and subsequently there has been legislative activity aimed at protecting retail investors. We find references to GetBack in all parliamentary materials associated with statutes passed after the commencement of the scandal. It is interesting to see that the impact of such a discrete event is statistically detectable. One might be tempted to draw parallels with how the Enron scandal in the U.S. resulted in the imposition of major compliance costs on publicly listed companies in the Sarbanes–Oxley Act of 2002 [27].

As regards the main subcomponents of the CRR hypothesis (H2 and H3), our data provides almost no evidence in favor of rejecting the null. Securities laws do not seem to be aimed at reducing transaction costs or risk to professionals any more than do statutes in general. Legislative intent with respect to those two matters seems to fluctuate to a considerable extent in either direction, depending on the subject matter of a given statute.

This gives rise to the following question: why is the CRR hypothesis so widely cited in the literature given that it does not appear to faithfully describe legislative intents? We see at least two *prima facie* plausible explanations. First, legal rules vary systematically among legal traditions [28]. The CRR hypothesis was formulated in the context of U.S. law, while our study was based on Polish law. It may be that the CRR hypothesis is well-supported by American federal securities law; that could be of interest for future research. Second, American scholars have particular affinity with the Law & Economics approach, while in Europe the prevalent method of legal scholarship is doctrinal, or “black letter law” [29,30]. Those who adopt the Law & Economics approach, which has traditionally been dominated by free-market considerations, are perhaps more likely to accept the CRR hypothesis.

The results show that the goals of securities laws, as reflected in legislative intents, vary in time significantly. Specific considerations appear to rise and fall over time in reaction to events that occur in society, such as financial scandals. A survey of legislative intents might thus serve as a useful barometer of social developments. For example, by using a different combination of research questions for the first stage of our research design (or by modifying them), one may investigate whether (and to what extent) ESG issues manifest themselves in specific areas of law. With the rise of ESG investing [8], securities laws may well be at the front of these developments. Indeed, upon closer inspection, we find that the newest securities statute (No. 1073) has been adopted in order to promote long-term investment horizons and improve non-financial performance of companies, including as regards ESG factors. We believe that this is a trend that may also be reflected in other areas of law, such as corporate law with its shareholder primacy doctrine. It would be interesting to see whether a trend like this is statistically detectable as a variation in legislative intent.

### Limitations

The two most important limitations of our study are the small sample of securities statutes and the lower number of experts surveyed. The small sample size is primarily due to the high concentration of Polish securities law in a small number of statutes, and partial harmonization of securities law at the EU level. The first issue could be addressed by including, in addition to statutes, regulations (*rozporządzenie*) of which around 1500–1800 were adopted annually in 2014–2019 (the majority of those was not related to securities law). The problem is that regulations are not subject to public consultation and, therefore, there is scant material concerning the legislative intent behind them. The second issue could be addressed by including EU law in the analyses, but there would have been substantial limitations. First, the number of EU laws by far exceeds the number of national statutes. Over an average of 1,300 EU directives and regulations are passed each year [31] in comparison to around 150–200 statutes in Poland (these figures include all laws, not only securities laws). Second, EU law is heavily weighted towards certain policy sectors, most importantly agriculture, foreign relations, and social, environmental, and regional issues [31]. Including EU law in the analysis would have risked skewing overall trends and precluded, in essence, intra-EU cross-country comparability. If we took the intent behind EU directives and regulations to be the legislative intent in all EU member states, the intent would be almost identical everywhere.

The second limitation–low number of experts–had two primary reasons. First, we limited our outreach to legal scholars in academia (due to their relative impartiality). Perhaps a follow-up survey among policymakers or other knowledgeable stakeholders could attract a larger number of respondents and reveal additional insights. Second, potential experts were generally reluctant to commit to such an expansive study. In order to score all statutes, an expert would have to pore through over 3,400 pages of abstracts (we have managed to find one such person), and even a single year required going through at least 500 pages. This is a particular challenge of our research design, but one that appears inevitable for securing accurate scoring.

Finally, the scores assigned to each statute are self-reported data and, therefore, could be influenced by a variety of cognitive biases, such as distinction bias, anchoring, or framing effects.

## Conclusion

A widely held view in the law and economics literature states that securities law aims at reducing transaction costs and risk to professional investors rather than at protecting consumers (the CRR hypothesis). Based on a survey of Polish statutory law, we find only partial support for the CRR hypothesis: securities laws are not, generally, enacted in order to ensure consumer protection. However, we find no compelling evidence in favor of the proposition that transaction costs and risk reduction are significant drivers of securities lawmaking. Changes in securities laws are not consistently intended to promote these goals.

The CRR hypothesis has a normative aspect, namely, it is based on the hidden assumption that market efficiency is more desirable than consumer protection. This assumption could potentially affect policy decisions that promote convergence in securities laws on regional or global levels, similarly to how the doctrine of shareholder primacy was meant to lead to global convergence on corporate law [12,13]. By showing that the CRR hypothesis does not hold universally across time and across borders, we hope to indirectly encourage lawmakers and regulators to carefully assess current local factors before they set policy in areas governed by securities laws.

## Supporting information

S1 Data (XLSX)
